# Recycling: Building on Fly Ash Waste

**Published:** 2007-01

**Authors:** David J. Tenenbaum

The quantity of fly ash—a waste product from coal smoke—is growing along with the steady global increase in coal use. According to Obada Kayali, a civil engineer at the University of New South Wales (UNSW) Australian Defence Force Academy, only 9% of the 600 million tons of fly ash produced worldwide in 2000 was recycled; most of the rest was landfilled. Now Kayali and colleagues have patented a technique for converting fly ash into Flashag™, an aggregate that can be mixed with sand, water, and portland cement to make concrete.

Flashag is made by heating fly ash until it crystallizes. In a presentation at the April 2005 World of Coal Ash Conference, Kayali said the patented process produces irregularly shaped aggregate that makes concrete with 25% more compressive strength than concrete made with fly ash pellets. Kayali and co-inventor Karl Shaw, also of UNSW, say Flashag concrete is also 21% lighter and up to 21% stronger than conventional concrete.

The higher strength is largely due to the presence of tiny craters on the aggregate, which allow the cement to attain a stronger bond. Stronger concrete means less is needed for a given application, reducing building weight, the quantity of material needed, and the energy used in transportation and handling. And thinner building components leave more space for occupancy.

Greater strength may also translate into fewer greenhouse gases, a major by-product of the high temperatures required for portland cement production. “A rough estimate yielded a possible reduction in greenhouse gas emission by around twenty-two percent,” Kayali says. Cost estimates for the Flashag process have not been worked out; what is known is that the process converts a major waste stream into a salable material.

A second UNSW patent involves heating a mix of fly ash, water, and a plasticizer to make bricks and other building materials. Only about two-thirds as much energy would be needed to make a brick that supports the same load as a standard clay brick.

Fly ash is not considered hazardous waste under the U.S. Resource Conservation and Recovery Act, and its composition can vary. But one primary constituent is carcinogenic silica, which can cause lung disease if inhaled. According to a study in the April 1987 *Water Resources Bulletin*, fly ash can also contain the toxic metals cadmium, copper, chromium, nickel, lead, mercury, titanium, arsenic, and selenium. However, tests showed no significant leaching from the new aggregate, according to Kayali and Shaw.

“This is an excellent idea,” says Tuncer Edil, who studies the reuse of coal combustion products at the University of Wisconsin–Madison. “Recycling fly ash, which is produced in large quantities, is important. It saves landfill space and cost, and alleviates the use of natural materials. It also leads to significant energy savings from crushing and transportation, and less environmental damage by quarrying. For the initial acceptance of Flashag, however, cost will be an important consideration, and current cost analyses do not account for certain environmental savings such as reduced emissions.”

